# Pre-Operative Calcitonin and CEA Values May Predict the Extent of Metastases to the Lateral Neck Lymph Nodes in Patients with Medullary Thyroid Cancer

**DOI:** 10.3390/cancers16172979

**Published:** 2024-08-27

**Authors:** Antonio Prinzi, Francesco Frasca, Marco Russo, Gabriella Pellegriti, Tommaso Piticchio, Dario Tumino, Antonino Belfiore, Pasqualino Malandrino

**Affiliations:** 1 Endocrinology, Department of Clinical and Experimental Medicine, University of Catania, Garibaldi-Nesima Medical Center, 95122 Catania, Italy; f.frasca@unict.it (F.F.); mruss@hotmail.it (M.R.); gabriella.pellegriti@unict.it (G.P.); tommaso.piticchio@phd.unict.it (T.P.); dariotumino@hotmail.com (D.T.); antonino.belfiore@unict.it (A.B.); p.malandrino@unict.it (P.M.); 2 Oncology, Department of Clinical and Experimental Medicine, University of Catania, 95123 Catania, Italy

**Keywords:** medullary thyroid cancer, calcitonin, CEA, neuroendocrine tumor, lymph nodes metastases

## Abstract

**Simple Summary:**

Total thyroidectomy and dissection of cervical lymph node compartments, depending on serum calcitonin levels and ultrasound findings, is standard treatment for patients with medullary thyroid cancer. The aim of this study was to evaluate whether pre-operative calcitonin and CEA levels can be useful as biomarkers of the extent of lymph node metastases at diagnosis. Results indicate that pre-operative serum calcitonin and CEA levels can predict presence, number, and site of lymph node metastases and, more specifically, values of 90 pg/mL for calcitonin and 17 ng/mL for CEA accurately indicate the N1b status. Since surgery is the only curative treatment for medullary thyroid cancer and there is not a strong indication regarding the extent of lymphadenectomy, these findings may help in the choice of the extent of neck dissection.

**Abstract:**

**Background**: In medullary thyroid cancer (MTC), lymph node metastases are often present at diagnosis and the extent of surgery is usually based upon pre-operative calcitonin and CEA levels as well as ultrasound findings. The aim of this study was to evaluate the role of pre-operative calcitonin and CEA levels as predictive markers of the burden of lymph node metastases at diagnosis. **Methods**: we conducted a retrospective study analyzing 87 MTC patients. **Results**: The median levels of calcitonin and CEA were 88.4 pg/mL and 7.0 ng/mL, respectively, in patients with no lymph nodes metastases; 108.0 pg/mL and 9.6 ng/mL, respectively, in patients with metastases to 1–5 lymph nodes; 520.5 pg/mL and 43.2 ng/mL, respectively, in patients with metastases to >5 lymph nodes. There were no significant differences in pre-operative calcitonin and CEA values between N0 and N1a patients, whereas they were significantly higher in N1b patients. Pre-operative cut-off levels distinguishing N0/N1a from N1b patients were 90 pg/mL for calcitonin (sensitivity 100%, specificity 59.3%, AUC = 0.82) and 17 ng/mL for CEA (sensitivity 100%, specificity 75%, AUC = 0.89). **Conclusions**: in patients with MTC, pre-operative serum calcitonin and CEA levels may drive the decision-making process to better define the extent of surgery.

## 1. Introduction

Medullary thyroid cancer (MTC) is a neuroendocrine tumor derived from the C cells of the thyroid gland that produce calcitonin (Ctn) [1,2]. The overall frequency of MTC is not well established, but there was an increase from 0.14 to 0.21 per 100,000 people in the United States population between 1983 and 2012 [3]. The prevalence of MTC is ~2% of all thyroid neoplasms and 0.4–1.4% of all thyroid nodules, and this tumor is detected in approximately 0.14% of thyroid samples obtained from autopsies [3]. MTC most commonly presents as a solitary thyroid nodule in patients in the fourth to sixth decade of life [4]. MTC occurs sporadically in 75% of patients. In the other 25% of patients, MTC is hereditary, mainly in the context of multiple endocrine neoplasia type 2A (MEN2A) (90–95%) and type 2B (MEN2B) (5–10%), which are autosomal dominant syndromes caused by a germline mutation in the REarranged during Transfection (*RET*) proto-oncogene [5].

The definitive cure of MTC is dependent on early detection and the completeness of the first surgical treatment [6]. Current guidelines recommend total thyroidectomy with central neck lymph node (CLN) dissection in patients with no evidence of neck lymph node metastases and no evidence of distant metastases. Dissection of the ipsilateral lateral compartments is recommended in patients with MTC confined to the central neck lymph nodes while contralateral lateral neck dissection should be considered if basal serum Ctn is greater than 200 pg/mL; however, the indications for prophylactic lateral neck lymph node (LLN) dissection and the recommended threshold levels of Ctn to warrant such intervention remain controversial [4]. Although the neck ultrasound (US) is a very reliable tool to detect metastatic lymph nodes, its accuracy is highly dependent on examiner’s experience [7] and micrometastases may be not correctly diagnosed. Moreover, the surgeon’s intraoperative assessment of lymph nodes may lead to misdiagnosing lymph node metastases (LNMs) in about 35% of excised nodal groups from the central or lateral compartments [8]. MTC can synthesize and secrete a variety of bioactive substances, such as Ctn and Carcinoembryonic Antigen (CEA), that, in addition to the neck US and intraoperative inspection, can be used like predictors of tumor extent in lymph nodes and eventually of the risk of recurrence [9,10,11,12]. Therefore, Ctn assay may be useful to suggest the most appropriate surgical intervention according to the pre-operative Ctn values. Previous studies reported that ipsilateral lateral LNM, contralateral lateral LNM, and distant metastasis were associated with pre-operative calcitonin thresholds of 20, 200, and 500 pg/mL, respectively [9,13]. In their study, Dr. Yip and coworkers observed that pre-operative Ctn values, but not pre-operative CEA, were correlated with tumor size [14]. At the opposite end, other studies reported that increased pre-operative CEA levels were associated with larger tumor size and the presence of lymph node and distant metastases [15,16]. Current guidelines recommend evaluating pre-operative Ctn values to consider the dissection of the ipsilateral and the contralateral lateral neck compartments [4]. However, no definitive recommendation on the usefulness of the CEA measure is provided.

The aim of this study was to evaluate whether pre-operative Ctn and CEA levels can be useful as biomarkers to gauge the extent of lymph node metastases (LNMs) at diagnosis, thus contributing to adequate pre-operative staging and providing useful indications to the surgeon when choosing the type of neck dissection.

## 2. Materials and Methods

### 2.1. Patients

**Figure 1:**
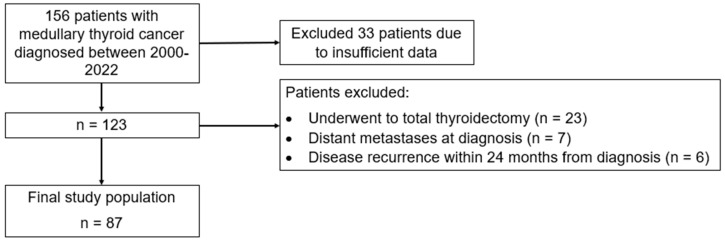
Flowchart depicting the selection process of the study cohort.

In this retrospective study, we analyzed data from a series of patients diagnosed with MTC who were referred to the outpatient Thyroid Clinic at the Garibaldi-Nesima Medical Center, a tertiary referral center (Endocrinology Unit, Department of Clinical and Experimental Medicine, Catania, Italy). In total, about 156 patients who were diagnosed with MTC at our Thyroid clinic between 2000 and 2022 were included in this study: of these, sufficient data were available for 123 patients. Among these, 116 patients were not affected by distant metastasis at diagnosis and only 87 underwent total thyroidectomy with neck dissection without evidence of disease recurrence for a minimum of 24 months and were selected for the purpose of this study (Figure 1). The retrospective study adhered to the Declaration of Helsinki and received approval from the institutional review board and Ethical Committee Catania 2 in Sicily, Italy. Signed informed consent from patients was waived due to the retrospective nature of this study. We obtained the medical records of all patients from our computerized database and selected them based on the following criteria: (1) underwent total thyroidectomy with lymph node dissection; (2) histologically diagnosed with MTC; (3) availability of pre-operative measurements for Ctn and CEA; (4) pre- and post-operative imaging assessment to evaluate disease extent; (5) complete resection of the disease through surgery (details below); (6) absence of distant metastases at the time of diagnosis and demonstrated no evidence of disease recurrence for a minimum of 24 months. Patients were considered free of disease after surgery if, for at least 24 months, they met the following criteria: both Ctn and CEA values remained below the normal upper limit, no lymph node metastases were identified through US features, confirmed by measuring Ctn in the washout from fine-needle aspiration biopsy of indeterminate lymph nodes, and no appearance of new lesions was suspected for distant metastases in computed tomography or magnetic resonance imaging.

### 2.2. Treatment and Follow-Up

According to the guidelines of the American Thyroid Association [4], the standard surgical treatment for MTC comprises total thyroidectomy and dissection of cervical lymph node compartments, depending on both serum Ctn levels and neck US findings. Patients without evidence of neck LNM had a total thyroidectomy with dissection of the lymph nodes in the bilateral central compartments. In the presence of LNM identified through US features and confirmed by measuring Ctn in the washout from the fine-needle aspiration biopsy of lymph nodes or in cases of high Ctn values (>200 pg/mL), dissection of lymph nodes in the lateral compartments was deemed appropriate; specifically, ipsilateral lateral neck dissection was performed in patients with MTC confined to central lymph node compartment (level VI), while contralateral lateral neck dissection was performed in patients with pre-operative LNM in the ipsilateral lateral neck or in case of pre-operative Ctn values > 200 pg/mL.

The first follow-up visit was performed 3 months after primary surgery, then every 6 months for the first years and yearly thereafter. The follow-up frequency varied based on the Ctn and CEA levels and the radiological findings.

### 2.3. Serum Markers and Pathological Examination

Ctn and CEA assays were performed pre-operatively in all patients. Serum Ctn was measured with the Siemens IMMULITE^®^ 2000 automatic chemiluminescence immunoassay analyzer using a standard assay kit for in vitro diagnosis (Siemens Healthcare Diagnostics Products Limited, Princeton, NJ, USA). CEA was measured with the Siemens Centaur^®^ XP automatic chemiluminescence immunoassay analyzer and its supporting reagents (Siemens Healthcare Diagnostics Inc.). Reference values were <10.0 pg/mL for Ctn and <5.0 ng/mL for CEA, in both males and females.

The histological samples were evaluated by an expert pathologist. The following features were recorded: size, multifocality, extrathyroidal extension, presence of LNM, and the number of metastatic lymph nodes. In multifocal or bilateral tumors, the largest tumor was considered.

### 2.4. Statistical Analysis

Quantitative data were presented as either the mean ± standard deviation (SD) or as the median and interquartile range (IQR) in cases of non-normally distributed variables. Qualitative data were expressed as numbers and percentages. Percentages were compared using the χ^2^ test. For comparing means between two groups of continuous variables, we employed the *t*-test, while the Kruskal–Wallis test was used for comparing means among more than two groups. Linear regression was employed to assess the relationship between continuous variables and other parameters, with logarithmic transformation applied to variables displaying non-normal distributions. Receiver operating characteristic (ROC) curve analysis was employed to determine the pre-operative Ctn and CEA cut-off levels that best predicted the presence of N1b. All analyses were performed using STATA version 17.0 (StataCorp, College Station, TX, USA). A *p*-value < 0.05 was considered statistically significant.

## 3. Results

### 3.1. Clinical and Histopathological Characteristics in Patients with MTC

We included 87 patients in this study. The mean age of the patients was 48.3 ± 18.6 years; 59 were women (67.8%) and 28 were men (32.2%). The median tumor size at diagnosis was 10 mm (25–75 IQR = 6–17 mm). Bilateral tumors were identified in 17 (19.5%) patients, while multifocal tumors occurred in 21 (24.1%) patients. The median follow-up duration was 91.0 months (25–75 IQR = 53.9–136.7). During this period, recurrent disease was observed in 10 patients: median time to progression = 105 months (25–75 IQR = 48–114).

A total of 60 (69%) patients received treatment with total thyroidectomy plus central lymph node dissection, while 17 (31%) patients underwent total thyroidectomy with both central and lateral ipsilateral and 10 with lateral bilateral neck dissection. We grouped the patients based on the lymph node status as follows: 70 (80.5%) patients showed no evidence of LNMs (N0), 11 (12.6%) patients had metastases in the central neck (N1a), while 6 (6.9%) patients had metastases in the lateral (N1b) neck compartments (3 ipsilateral and 3 bilateral) (Table 1). Most patients (*n* = 43, 49%) had sporadic MTC, negative for RET germline mutation. Additionally, 34 (39%) patients showed familial MTC due to a germline RET mutation, while in 10 (12%) patients, the RET status was unknown. Among patients with familial MTC, 22 mutations were found in exon 618, 5 in exon 804, 3 in exon 634, 1 in exon 649, 1 in exon 768, 1 in exon 791, and 1 in exon 790.

### 3.2. Pre-Operative Ctn and CEA Levels

**Figure 2:**
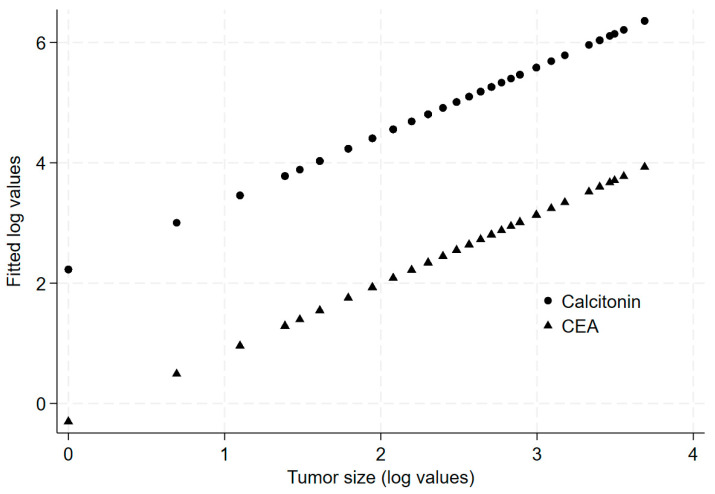
Predicted values of calcitonin and CEA based on tumor size.

Pre-operative Ctn and CEA values increased significantly as the tumor size increased (*p* < 0.001 by univariate linear regression) (Figure 2). According to the American Joint Committee on Cancer TNM Classification (Thyroid Cancer), cancers are classified into four groups based on their maximum diameter: ≤10 mm (T1a), 11–20 mm (T1b), 21–39 mm (T2), and >40 mm (T3). The median pre-operative Ctn levels were 60 pg/mL in the T1a group, 234 pg/mL in the T1b group, 630 pg/mL in the T2 group, and 739 pg/mL in the T3 group (Table 2). The median pre-operative CEA levels were 5.5 ng/mL in the T1a group, 17.9 ng/mL in the T1b group, 43.7 ng/mL in the T2 group, and 52.0 ng/mL in the T3 group (Table 2). Median Ctn levels were not associated with multifocality or bilaterality, and CEA values were not associated with bilaterality either. In contrast, CEA values were significantly lower in patients with multifocal MTC compared to those with unifocal MTC (2.60 vs. 14.87 ng/mL, respectively).

The median Ctn values were 88.4 (25–75 IQR = 27–282) and 118 (25–75 IQR = 68–448) pg/mL in patients without (N0) and with (N1) LNM, respectively (*p* = 0.043). The median CEA values were 7.0 (25–75 IQR = 1.9–28) and 17.9 (25–75 IQR = 2.6–53.8) ng/mL in N0 and N1 patients, respectively (*p* = 0.027). Compared to N0 patients, the median pre-operative Ctn values showed no significant difference in N1a patients (98.1 pg/mL; *p* = 0.59). However, the median pre-operative Ctn values were notably higher in N1b patients (520.5 pg/mL; *p* = 0.003). Similarly, the median pre-operative CEA values did not significantly differ in N1a patients (2.6 ng/mL; *p* = 0.48), but they were significantly elevated in N1b patients (53.8 ng/mL; *p* = 0.006). We then classified the patients according to the number of metastatic lymph nodes found at histology: 0, 1–5, >5: 70 (80.5%) patients had no LNMs, 13 (14.9%) patients had 1–5 LNMs, and 4 (4.6%) had >5 LNMs. The median Ctn and CEA levels were, respectively, 88.4 pg/mL and 7.0 ng/mL in patients with 0 LNMs, 108.0 pg/mL and 9.6 ng/mL in patients with 1–5 LNMs, and 520.5 pg/mL and 43.2 ng/mL in patients with >5 LNMs (Table 2). The difference between the three prognostic groups was statistically significant for both pre-operative Ctn levels (*p* = 0.017) and pre-operative CEA levels (*p* = 0.013).

### 3.3. Cut-Off Values of Pre-Operative Ctn and CEA Levels Predictive of the N1b Status

**Figure 3:**
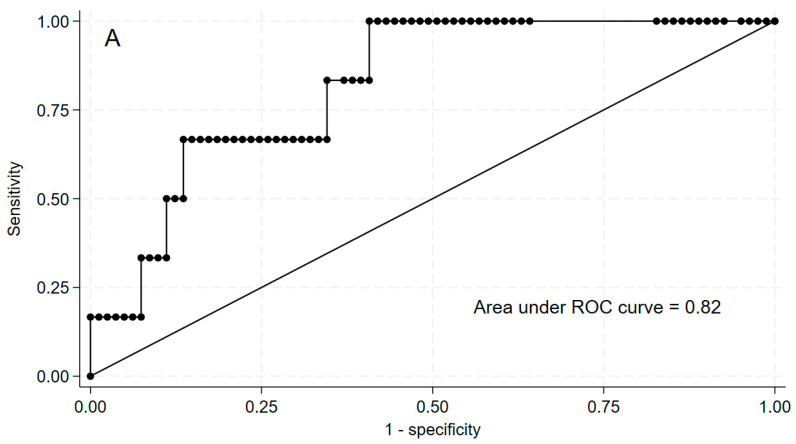

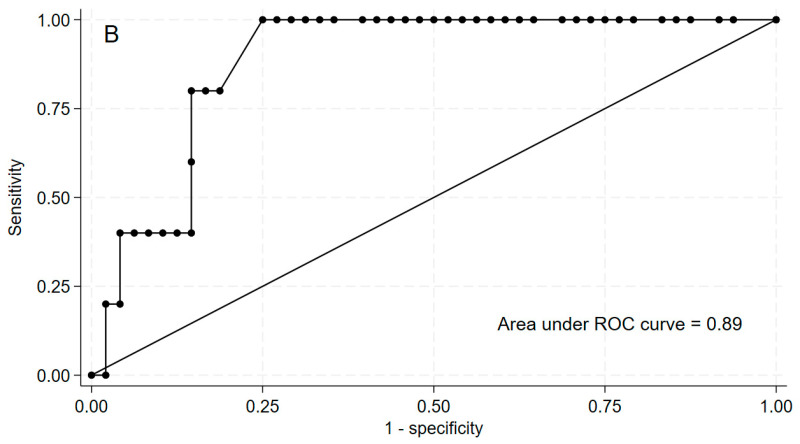
Comparison of pre-operative calcitonin (panel (**A**)) and CEA (panel (**B**)) receiver operating characteristic (ROC) curves considering patients with N1b metastases as positive controls.

We then used ROC curve analysis to estimate the cut-off values of pre-operative Ctn and CEA levels that best predicted the N1b status. The pre-operative Ctn value that best distinguished between N0/N1a and N1b patients was 90.0 pg/mL (sensitivity 100%, specificity 59.3%, positive predictive value 13.9%, negative predictive value 100%, area under the curve [AUC] = 0.82) (Figure 3). The corresponding pre-operative CEA value was 17 ng/mL (sensitivity 100%, specificity 75%, positive predictive value 18.2%, negative predictive value 100%, AUC = 0.89) (Figure 3). A pre-operative Ctn ≥ 90 pg/mL was observed in all patients with N1b and in 50% of patients with N0/N1a (*p* = 0.019). Similarly, a pre-operative CEA ≥ 17 ng/mL was observed in all patients with N1b and in only 30% of patients with N0/N1a (*p* = 0.016). So, no patients with pre-operative Ctn < 90 pg/mL and pre-operative CEA < 17 ng/mL had N1b metastases.

## 4. Discussion

Surgery is the only curative treatment in patients with MTC; it may allow a radical cure through the removal of all tumoral tissue and LNMs [17]. There is no strong indication regarding the extent of lymphadenectomy: central neck dissection is recommended because of the high frequency of occult LNMs in the central neck compartment. However, the indication for prophylactic excision of the LLN remains controversial. Patients with LNMs visible on US in the lateral compartments of the neck should undergo total thyroidectomy with central and lateral neck dissection [5]. Although a wider lymph node dissection may reduce the chance of recurrent disease, it adversely affects the patient’s quality of life. Indeed, an aggressive neck dissection can increase the risk of complications [18]. Transient or permanent hypoparathyroidism, recurrent laryngeal nerve injury or paralysis and hematoma are more frequently observed after central neck dissection, whereas seroma, chyle fistula, Horner’s syndrome, and motor nerve injury of the neck are more frequently observed after lateral neck dissection [19]. Although repeated surgery is feasible, it is burdened by a complication rate of 5–14% even in expert hands [20].

A series of pre-operative imaging procedures are currently used to accurately stage the tumor and to decide the most suitable extent of surgery. Neck US is the most important pre-operative imaging study; however, other radiological imaging procedures are used when metastatic disease is suspected: computed tomography (CT) is the most sensitive imaging procedure for detecting lung metastases and LNMs while magnetic resonance imaging (MRI) is the most sensitive method to detect liver metastases. 2-[Fluorine18]fluoro-2-deoxy-d-glucose (FDG)-positron emission tomography (PET) appears to be less sensitive and of low prognostic value in detecting metastases compared with the other imaging procedures [21], including 18F-DOPA PET [22] and 68Ga PET [23].

The aim of this study was to investigate the possible clinical application of serum Ctn and CEA values to predict the presence of metastases in the lateral neck of patients with MTC. To largely exclude the possibility that a patient who underwent thyroidectomy plus central neck dissection could have metastases to the lateral neck, we selected patients with no evidence of biochemical or structural disease within 24 months after surgery. We believe that this is a reasonable interval since, according to a recent study, the median time to recurrence in patients with MTC was approximately 2.1 years [24]. Pre-operative basal Ctn has a well-established role as a marker for pre-operative diagnosis and post-surgical follow-up [25,26,27,28]. Serum CEA is widely used as tumor marker for breast, gastrointestinal, and lung cancer [28,29,30,31]. The first study that proposed using serum CEA for follow-up in MTC dates back to more than 30 years ago [32,33]. Today, it is widely used in the post-surgical follow-up.

In this study, we found that the Ctn levels correlate with tumor size, the number of LNMs, and the presence of LNMs in the lateral neck. Likewise, the pre-operative basal CEA levels could be used as an indicator of tumor size and the number of LNMs and could predict the presence of LNMs in the lateral neck. Machens and Dralle [9] reported LNMs in the ipsilateral central and lateral neck, contralateral central neck, contralateral lateral neck, and upper mediastinum when basal Ctn levels were above the threshold levels of 20, 50, 200, and 500 pg/mL, respectively. We found that no patients had N1b metastases when pre-operative Ctn levels were <90 pg/mL (AUC = 0.82). As expected, our determined Ctn threshold varies from that identified by Machens and Dralle due to inherent challenges in Ctn measurement, particularly concerning preanalytical phases and interassay comparability. Consequently, a cut-off value established for one assay might not be universally applicable to others. In clinical practice, it is imperative to account for the specific assay employed and its validated cut-off values, rather than relying exclusively on generalized thresholds. Indeed, the current guidelines do not specify a reference range of basal or stimulated serum Ctn levels that may allow a diagnosis of MTC or exclude it and recommend using certain reference ranges established by individual laboratories [4].

Another study evaluated the role of pre-operative CEA for detecting LNM before surgery [34]. The authors reported that CEA levels are related to the presence of lateral LNM and the cut-off to predict the presence of lateral LNM was 29.68 ng/mL (AUC: 0.831). In the present study, we found that LNMs in the lateral neck were present when pre-operative CEA was ≥17.0 ng/mL (AUC = 0.89). Interestingly, no patients had N1b metastases below this value. Combining this observation with the identified Ctn cut-off, we demonstrate that both measurements may serve as a rule out test for accurately excluding the presence of N1b in patients with MTC. These cut-off values could help the surgeon during pre-operative planning to choose the correct type of surgery, avoiding aggressive neck dissection when there is no need and thus sparing patients from potential post-surgical complications.

Procalcitonin may serve as a dependable alternative tumor marker in MTC for both prognostication and treatment monitoring, exhibiting comparable performance to Ctn [35]. Interestingly, procalcitonin proved being a reliable marker to distinguish between the presence or absence of structural disease during follow-up due to a high negative predictive value [36]. However, owing to its superior preanalytical and analytical characteristics, procalcitonin holds significant potential to replace Ctn as the new standard of care in managing MTC. Another promising marker for predicting the extent of disease at diagnosis is the Calcitonin Secretory Index (CSI); in the study of Filimon et al., the authors showed that CSI may predict early disease extent and persistent disease [37].

This study has some limitations as it deals with a retrospective analysis of a series of patients recruited at a single center. For this reason, we had no data on procalcitonin values in our patients with MTC. Moreover, our results may not be generalizable to other clinical settings because a cut-off value established for one assay might not apply to another due to differences in the preanalytical phase and interassay comparability. Therefore, in clinical practice, it is crucial to consider the specific assay used and its validated cut-off values rather than relying solely on generalized cut-offs. The data appear potentially important for the clinical management of patients with MTC, but additional research involving a larger number of N1b patients is needed to validate these results. Moreover, there is a need for standardization programs and comparative studies aimed at harmonizing Ctn and CEA assays and establishing universally applicable cut-off value guidelines.

## 5. Conclusions

In patients with MTC, pre-operative serum Ctn and CEA levels can predict the presence, number, and site of LNMs, providing useful information that, in addition to imaging techniques, may help in the choice of the extent of neck lymph node dissection.

## Figures and Tables

**Table 1:** Clinicopathological features at diagnosis of 87 patients with medullary thyroid cancer (MTC).

Parameter	N = 87
Age, years (mean ± SD)	48.3 ± 18.6
Gender, female *n*. (%)	59 (67.8)
Sporadic MTC, *n*. (%)	52 (59.8)
Tumor size (mm), median (25–75 IQR)	10 (6–17)
Total thyroidectomy with central LN dissection, *n*. (%)	60 (69)
Total thyroidectomy with both central and lateral LN dissection, *n*. (%)	27 (31)
Bilateral tumor, *n*. (%)	17 (19.5)
Multifocal tumor, *n*. (%)	21 (24.1)
N0, *n*. (%)	70 (80.5)
N1a, *n*. (%)	11 (12.6)
N1b, *n*. (%)	6 (6.9)
LN = lymph node	

**Table 2:** Median value of serum calcitonin (Ctn) and CEA based on tumor size number of metastatic lymph node and N category.

Tumor Size (mm)	Ctn Value (Median and IQR Range, pg/mL)	CEA Value (Median and IQR Range, ng/mL)
≤10	60 (24–94)	5.5 (1.3–7)
11–20	234 (125–472)	17.9 (13.9–50.7)
21–39	630 (503–722)	43.7 (28–67.1)
≥40	739 (447–1189)	52.0 (37.6–59.9)
**Number of metastatic lymph node**		
0	88.4 (27–282)	7.0 (1.9–28)
1–5	108.0 (57.4–269)	9.6 (1.8–35.2)
>5	520.5(272–657.5)	43.2 (19.2–67.1)
**Lymph node status**		
N0	88.4 (27–282)	7 (1.9–28)
N1a	98.1 (57.4–145)	2.6 (1–16.5)
N1b	520.5 (118–722)	53.8 (19.2–67.1)

## Data Availability

The data presented in the following study are available from the corresponding authors upon request.
